# Supplementary material for: Comparison of the Safety and Immunogenicity of FAKHRAVAC and BBIBP-CorV Vaccines when Administrated as Booster Dose: A Parallel Two Arms, Randomized, Double Blind Clinical Trial
Source: Vaccines (Basel). 2022 Oct 26;10(11):1800. doi: 10.3390/vaccines10111800 (PMC9695457; doi:10.3390/vaccines10111800)
Supplement: Supplementary file 1 [file vaccines-10-01800-s001.zip › Supplementary File S1 - Clinical Trial Protocol.pdf]

Clinical Trial Protocol
Iranian Registry of Clinical Trials

12 Jun 2022

Comparison of safety and immunogenicity of FAKHRAVAC and Sinopharm booster doses for adults 18 years of age and older, fully vaccinated by Sinopharm: a parallel 2 arms, randomised, double blind clinical trial

Protocol summary

Study aim

Comparison of safety and immunogenicity of FAKHRAVAC and Sinopharm booster dose for adults 18 years of age and older, fully vaccinated by Sinopharm

Design

Randomized, double blind, controlled trial with parallel group design on 400 volunteers in 2 groups of 200, stratified on for time periods since the last dose of primary vaccination (75-105, 106-135, 136-165, 166-195 days).

Settings and conduct

SASAD Sports Complex, Shahid Fakhrizadeh Street, Sayad Shirazi Highway

Participants/Inclusion and exclusion criteria

Inclusion criteria: Age more than 18 years; Not having COVID 19 since the last dose of primary vaccination with Sinopharm; Fully vaccinated and within the 75 to 195 days post vaccination period; Signing the informed consent form. Main exclusion criteria: Current acute or chronic symptomatic illness that requires ongoing medical or surgical care; History of severe cardiovascular disease; Pregnancy and lactation.

Intervention groups

Intervention group1: One dose of FAKHRAVAC vaccine injected in the deltoid muscle (IM) Intervention group2: One dose of Sinopharm vaccine injected in the deltoid muscle (IM)

Main outcome variables

Primary outcome: Neutralizing antibody activity 2 weeks after the booster dose Secondary outcomes: Abnormal vital signs and anaphylactic reactions immediately after vaccination; Local and systemic reactions within the first week after booster dose; Serum ELISA IgG level for SARS-CoV-2 N, S1-RBD antigens; SAEs, SUSARs, MAAEs, up to a month following the booster dose; Neutralizing antibody activity and serum ELISA IgG level for SARS-CoV-2 N, S1-RBD antigens, 3 and 6 months after the booster dose (in a subgroup of participants).

General information

Reason for update

Acronym

IRCT registration information

IRCT registration number: **IRCT20210206050259N4**
Registration date: **2021-11-29, 1400/09/08**
Registration timing: **prospective**

Last update: **2021-11-29, 1400/09/08**
Update count: **0**

Registration date

2021-11-29, 1400/09/08

Registrant information

Name

Ahmad Karimi Rahjerdi

Name of organization / entity

Milad Darou Noor Parphaceutical Company (MDNP)

Country

Iran (Islamic Republic of)

Phone

+98 912 722 0594

Email address

A-Karimi@mdnp.ir

Recruitment status

Recruitment complete

Funding source

Expected recruitment start date

2021-12-01, 1400/09/10

Expected recruitment end date

2022-03-01, 1400/12/10

Actual recruitment start date

empty

Actual recruitment end date

empty

Trial completion date

empty

Scientific title

Comparison of safety and immunogenicity of FAKHRAVAC and Sinopharm booster doses for adults 18 years of age and older, fully vaccinated by Sinopharm: a parallel 2 arms, randomised, double blind clinical trial

Public title

Comparison of safety and immunogenicity of FAKHRAVAC and Sinopharm booster doses for adults 18 years of age and older, fully vaccinated by Sinopharm

Purpose

Prevention

Inclusion/Exclusion criteria

Inclusion criteria:

Age more than18; Not having COVID 19 since the last dose of primary vaccination with Sinopharm; Fully vaccinated and within the 75 to 195 days post vaccination period; Signing the informed consent form; For females of childbearing age 18 to 49 years: use of at least one effective method of contraception (condom, oral contraceptive pills, intrauterine device, norplant capsule) and willing to continue up to two month after the booster dose.

Exclusion criteria:

History of allergy to drugs or vaccines (e.g urticaria and fever); Current acute or chronic symptomatic illness that requires ongoing medical or surgical care; History of severe cardiovascular disease; Lactation; History of receiving any vaccine during the 14 days period prior to the day of receiving booster dose; History of transfusion of any blood product or immunoglobulin within the 3 months period before receiving booster dose; History of diseases resulting in immunosuppression (suspected and definite); History of long-term use of immunosuppressive drugs or systemic corticosteroids in the last 4 months period leading up to the screening day; History of diagnosis or treatment for cancer (except basal cell carcinoma and Insitu cervical cancer); History of uncontrolled serious psychiatric illnesses; History of blood disorders (Blood Dyscrasias, coagulation disorders, platelet deficiency, etc); History of chronic neurological diseases (including seizure and epilepsy); Current drug/alcohol abuse (addiction); Acute febrile illness at the time of booster vaccine injection; Having splenectomy for any reason; Any close contact with a definitively infected person with COVID-19 within the two weeks period before the day of receiving the booster dose; Current use of anticoagulants such as coumarin and related anticoagulants (such as warfarin) or new oral anticoagulants / antiplatelet agents. Note: Less than 325 mg of aspirin per day as prophylaxis is allowed; Chronic unstable disease (last 4 weeks) at the discretion of the principal investigator; Pregnancy.

Age

From **18 years** old

Gender

Both

Phase

N/A

Groups that have been masked

- Participant
- Care provider
- Investigator
- Outcome assessor
- Data and Safety Monitoring Board

Sample size

Target sample size: **400**

Randomization (investigator's opinion)

Randomized

Randomization description

In this study, stratified block randomization method with block size of 4 was used to assign each participant to the intervention groups. The rand() function of Excel software were used to generate random sequence within each block. After determining the allocated intervention, a non-repetitive five-digit random code was assigned to each participant.

Blinding (investigator's opinion)

Double blinded

Blinding description

In this study, the control group will receive the Sinopharm vaccine, which has different packaging and shape compared to FakhraVac. Therefore, implementation of blinding will be done by a person who will be responsible for this. This is the only person who will not be blind to the intervention given. Once the participant becomes eligible to receive the vaccine, a concealment/randomization code will be assigned to the volunteer and the vaccine type will be displayed on the screen of the vaccinator until the inoculation is confirmed.

Placebo

Not used

Assignment

Parallel

Other design features

Secondary Ids

empty

Ethics committees

1

Ethics committee

Name of ethics committee

National Research Ethics committee

Street address

Floor 13, Block A, Ministry of Health & Medical Education Headquarters, Between Zarafashan & South Falamak, Qods Town, Tehran, Iran

City

Tehran

Province

Tehran

Postal code

7334144696

Approval date

2021-11-28, 1400/09/07

Ethics committee reference number

IR.NREC.1400.014

Health conditions studied

1

Description of health condition studied

Respiratory Distress Syndrome due to SARS-CoV-2

ICD-10 code

U07.1

ICD-10 code description

ICD-10COVID-19, virus identified

Primary outcomes

1

Description

Neutralizing antibody activity

Timepoint

On day zero, two weeks, 3 and 6 months after the booster dose injection

Method of measurement

SARS-CoV-2 virus neutralizing antibody titter measured in bio-safety level III lab using conventional method.

Secondary outcomes

1

Description

Abnormal vital signs and anaphylactic reactions immediately after vaccination

Timepoint

In the first half an hour after the booster dose

Method of measurement

Temperature is measured using a digital thermometer. Respiratory rate will be counted by the research staff over one minute. Blood pressure and heart rate will be measured by a digital sphygmomanometer in a sitting position.

2

Description

Local adverse reactions within the first week after booster dose

Timepoint

Daily, within the first week after the booster dose

Method of measurement

Via mobile application, study staff will contact participants who fail to fill their application and complete a local adverse reaction form on their behalf.

3

Description

Systemic adverse reactions within the first week after booster dose

Timepoint

Daily, within the first week after booster dose

Method of measurement

Via mobile application, study staff will contact participants who fail to fill their application and complete a systemic adverse reaction form on their behalf.

4

Description

SAEs, SUSARs, MAAEs, up to 1 month after the booster dose

Timepoint

Up to one month after the booster dose

Method of measurement

Via mobile application. There will be a 24-7 followup center with physicians available all the time.

5

Description

Serum ELISA IgG level for SARS-CoV-2 N, S1-RBD antigens

Timepoint

On day zero, two weeks, 3 and 6 months after the booster dose vaccine

Method of measurement

ELISA method

Intervention groups

1

Description

Intervention group1: One dose of FAKHRAVAC vaccine injected in the deltoid muscle (IM) Intervention

Category

Prevention

2

Description

Intervention group2: One dose of Sinopharm vaccine injected in the deltoid muscle (IM)

Category

Prevention

Recruitment centers

1

Recruitment center

Name of recruitment center

Fakhra clinical trial center

Full name of responsible person

Mohsen Forughizadeh Moghadam

Street address

Fakhra clinical trial center, Persian Gulf Hall, SASAD Sports Complex, Shahid Fakhrizadeh Street, Sayad Shirazi Highway, Tehran, Iran

City

Tehran

Province

Tehran
Postal code
1986936911
Phone
+98 21 2610 1694
Email
Foroughizadeh@modares.ac.ir

Sponsors / Funding sources

1

Sponsor
Name of organization / entity
Organization of Defensive Innovation and Research
Full name of responsible person
Ahmad Karimi Rahjerdi
Street address
NO.9, Unit 3, Mirsharifi, Valiasr St.
City
Tehran
Province
Tehran
Postal code
1986936911
Phone
+98 21 2265 8405
Email
A-Karimi@mdnp.ir
Grant name
Grant code / Reference number
Is the source of funding the same sponsor organization/entity?
No
Title of funding source
Organization of Defensive Innovation and Research
Proportion provided by this source
100
Public or private sector
Public
Domestic or foreign origin
Domestic
Category of foreign source of funding
empty
Country of origin
Type of organization providing the funding
Other

Person responsible for general inquiries

Contact
Name of organization / entity
Malek Ashtar University
Full name of responsible person
Mohsen ForughiZadeh Moghadam
Position
Assistant professor
Latest degree
Ph.D.
Other areas of specialty/work
Medical Genetics
Street address
Malek Ashtar University, Shabanloo St., Lavizan
City
Tehran
Province
Tehran
Postal code
1955737134
Phone
+98 21 8008 6783
Email
Foroughizadeh@modares.ac.ir

Person responsible for scientific inquiries

Contact
Name of organization / entity
Artesh University of Medical Sciences
Full name of responsible person
Ramin Hamidi Farahani
Position
Associate professor
Latest degree
Specialist
Other areas of specialty/work
Infectious diseases
Street address
NO.9, Unit 3, Mirsharifi, Valiasr St.
City
Tehran
Province
Tehran
Postal code
1986936911
Phone
+98 21 8833 7912
Email
Rgsramin@yahoo.com

Person responsible for updating data

Contact
Name of organization / entity
Milad Daro Noor Pharmaceutical Co.
Full name of responsible person
Kosar Naderi
Position
Assistant Professor
Latest degree
Ph.D.
Other areas of specialty/work
Biology
Street address
NO.9, Unit 3, Mirsharifi, Valiasr St.
City
Tehran
Province
Tehran
Postal code
1986936911
Phone
+98 21 2265 8405

Email
k.naderi@strc.ac.ir

Sharing plan

Deidentified Individual Participant Data Set (IPD)
Yes - There is a plan to make this available
Study Protocol
Yes - There is a plan to make this available
Statistical Analysis Plan
Yes - There is a plan to make this available
Informed Consent Form
Yes - There is a plan to make this available
Clinical Study Report
Yes - There is a plan to make this available
Analytic Code
Yes - There is a plan to make this available
Data Dictionary
Not applicable
Title and more details about the data/document
Deidentified IPD on study outcomes could be shared.
When the data will become available and for how long
After completion of the study and publication of the results, data could be shared for 2 years
To whom data/document is available
Data is available only to members of academic institutions within joint projects with MILAD Daru Nour Co
Under which criteria data/document could be used
Proposal should be presented to MILAD Daru Nour Co. A scientific Advisory committee to MILAD Daru Nour Co should confirm necessity and scientific validity of the proposed joint project
From where data/document is obtainable
You can contact Ms Kousar Naderi at k.naderi@strc.ac.ir
What processes are involved for a request to access data/document
Request for data will be made available within the approved joint projects
Comments
