# Supplementary material for: Comparison of the Safety and Immunogenicity of FAKHRAVAC and BBIBP-CorV Vaccines when Administrated as Booster Dose: A Parallel Two Arms, Randomized, Double Blind Clinical Trial
Source: Vaccines (Basel). 2022 Oct 26;10(11):1800. doi: 10.3390/vaccines10111800 (PMC9695457; doi:10.3390/vaccines10111800)
Supplement: Supplementary file 1 [file vaccines-10-01800-s001.zip › Supplementary File S2 - Consent form booster.pdf]

Consent form of participation in the study on "comparison of safety and immunogenicity of FAKHRAVAC and Sinopharm booster doses for adults 18 years of age and older, fully vaccinated by Sinopharm: a parallel two arms, randomized, double-blind clinical trial."

I know that the main purpose of this research is to evaluate the safety and immunity of a booster dose of the Fakhra vaccine with a booster dose of Sinopharm vaccine in adults 18 years and older vaccinated with the Sinopharm vaccine. Covid 19 disease, which has infected almost every country and region in the world, is currently not effectively treated. The Fakhra vaccine used in this study is new, and in phase 1, adults to 135 healthy volunteers, and in phase 2, adults to 500 healthy people aged 18 to 70 years or with some diseases (Has been injected and has been used on 15,000 volunteers in phase three so far, and in addition to not causing any severe or profound side effects, it has also been shown to have good immunogenicity. The World Health Organization also approved the Sinopharm vaccine used in this study.

This study answers whether the harmlessness and immunogenicity of this new vaccine called Fakhra as a booster dose are equal to the Sinopharm vaccine (a vaccine approved by the World Health Organization). In this study, people vaccinated with the Sinopharm vaccine received a dose of Covid-19 vaccine as a booster dose, and the vaccine was randomly assigned to one of the following two groups:

Group 1: Participants in this group receive one dose of FAKHRAVAC vaccine as a booster dose as IM in the deltoid muscle.

Group 2: Participants in this group receive a single dose of Sinopharm vaccine as a booster dose as IM in the deltoid muscle.

During the study period, information about possible symptoms after receiving the vaccine is collected in person or in-person as needed.

I was explained that more information about this research could be found on the Iranian Clinical Trial Registration Center website at http://www.irct.ir using the registration code for this study at IRCT20210206050259N4.

I know my participation in this research is entirely voluntary, and I do not have to participate.

I was assured that if I refused to participate in this study, I would not have an unpleasant outcome regarding receiving health services.

I know that even after agreeing to participate in the research, I can leave the stud whenever I want after informing the facilitator. My withdrawal from the research will not deprive me of receiving the usual health care services. I was informed that if I left the study, I would not be notified of the group I was in until the end of the study unless requested by the Safety and Data Monitoring Committee.

I was informed that the way I cooperated in this study was that after I consented to participate in the study, first the initial examinations, including medical records, examinations, and diagnostic tests (blood sample for immunological tests), A urine test will be performed to diagnose possible pregnancy in women). Suppose I meet the conditions for inclusion in the study. In that case, I will be injected with a dose of the Fakhra vaccine produced by Milad Daroo Noor Company or Sinopharm vaccine randomly and without any choice on my part intramuscularly in the deltoid muscle of the arm. I consider myself obligated and committed to comply with all of the following:

- In this study, I will receive a dose of vaccine by intramuscular injection in the deltoid muscle of the arm. After administering the vaccine, I will be monitored for 30 minutes to ensure complete health.

- During the study process, I may need to have tests, and I agree to cooperate fully with the research team to receive the vaccine and perform medical tests.

- I am supposed to be given forms regarding the registration of tolls, which I must complete exactly if a possible toll is found and submit on time.

- I should not donate blood throughout the study and not participate in another clinical trial. Also, notify the research team if I have to use another drug or vaccine or need to be hospitalized for any reason.

- All stages of the study, including the initial examination, tests, vaccination, and subsequent examinations and visits, are performed at the site introduced by the study agents.

I was informed that the potential benefits of my participation in this study are as follows:

I will receive a dose of Covid 19 injectable vaccine produced by Milad Daroo Noor Company or Sinopharm vaccine intramuscularly.

I was taught that if I was exposed to the virus in the future, I should not think that because I received the booster vaccine in this study, it would protect me 100% against Covid-19.

I was taught that the possible harms and complications of participating in this study are as follows:

Due to the harmless approval of the vaccine produced by Milad Daroo Noor Company in phases 1 and 2 and the approval of the Sinopharm vaccine by the World Health Organization and the Ministry of Health, the possibility of harm and side effects following the vaccine is low. However, any drug or biological product may have side effects. Be; Including pain, redness, swelling, or bruising at the injection site, as well as mild symptoms such as fever, headache, fatigue, and body aches in the first 48 hours or up to a week after the injection. These symptoms are expected to be mild if they do occur, but there may be moderate to severe symptoms.

If I do not want to participate in the study, I will not have an unpleasant consequence regarding receiving health care services.

I know that those involved in this research have kept all information related to my health record confidential and are only allowed to publish the general and group results without mentioning my name and details.

I have been informed that the Research Ethics Committee may have access to the information recorded in this study in order to monitor compliance with my rights.

I know that I will not be responsible for any costs incurred in the research project, including the cost of examinations and tests, and that the study sponsor will bear any compensation related to the vaccine. I was informed that the sponsor of this study is Milad Daroo Noor Company. This company will cover all volunteers during the study. In case of any damage related to the vaccine, the relevant costs will be fully compensated by this company.

I was given telephone numbers to answer, and I was told to share any problems or questions about participating in the study with them and ask for help. In this regard, I agree that telephone calls made to

physicians and clinical associates regarding questions and possible vaccine side effects should be recorded to record research information accurately.

Landline: 021-22984052 Mobile: 09033021285

I know that if, during and after the research, any problem, both physical and mental, occurred to me due to participating in this research, the treatment of its complications and costs and the appropriate compensation will be the responsibility of the executor.

I know that if I have any problems or objections to those involved or the research process, I can contact the National Committee for Research Ethics in Tehran. Phone: 021-81455618 and raise my problem orally or in writing.
