# Supplementary material for: Comparison of the Safety and Immunogenicity of FAKHRAVAC and BBIBP-CorV Vaccines when Administrated as Booster Dose: A Parallel Two Arms, Randomized, Double Blind Clinical Trial
Source: Vaccines (Basel). 2022 Oct 26;10(11):1800. doi: 10.3390/vaccines10111800 (PMC9695457; doi:10.3390/vaccines10111800)
Supplement: Supplementary file 1 [file vaccines-10-01800-s001.zip › Supplementary File S3 - Informed Consent.pdf]

Informed consent form

Comparison of the safety and immunogenicity of FAKHRAVAC and BBIBP-CorV vaccines when administrated as booster dose: A parallel two arms, randomized, double blind clinical trial

1. I know that the aim of this research is:

The Covid-19 disease, which has infected almost all countries and regions of the world, currently has no effective treatment. The Fakhra vaccine that is used in this study is new, and in the 1st phase of adults, it was given to 135 healthy volunteers, and in the 2nd phase of adults, it was given to 500 healthy people between the ages of 18 and 70 or suffering from some diseases. controlled blood pressure and diabetes) has been injected, and so far, it has been used on 15,000 volunteers in phase three; in addition to the fact that no severe and dangerous complications related to it have been observed, it has also had good immunogenicity. The World Health Organization approved the Sinopharm vaccine used in this study.
This study answers whether the harmlessness and immunogenicity of this new vaccine called Fakhra as a booster dose are equal to the Sinopharm vaccine (vaccine approved by the World Health Organization). In this study, the people vaccinated with the Sinopharm vaccine as a booster dose will receive one round of the Covid-19 vaccine, and the people will be randomly assigned the vaccine to one of the following two groups:

- Group 1: The participants of this group receive one dose of the FAKHRAVAC vaccine as a booster dose of IM in the deltoid muscle.
- Group 2: The participants of this group receive one dose of Sinopharm vaccine as a booster dose of IM in the deltoid muscle.

During the study period, information on possible symptoms after receiving the vaccine will be collected in person or person as needed.
It was explained to me that I could search and view more information about this research on the website of the Iranian Clinical Trial Registration Center at www.irct.ir using the registration code of this study (Registration code: IRCT20210206050259N4).

2. I know my participation in this research is voluntary, and I do not have to participate.
3. I was assured that if I refused to participate in this study, I would not be disadvantaged in receiving health care.
4. I know that even after agreeing to participate in the research, I can leave the study whenever I want after informing the research team.
5. The method of my cooperation in this study is as follows: after announcing my consent to participate in the study, I will be examined by a qualified doctor. This will include interviews, clinical examinations, psychiatric assessments, and giving samples for diagnostic tests (blood and urine). If I meet the eligibility requirements to enter the study, I will then be invited to attend the main stage of the study, and the FAKHRAVAC vaccine produced by Milad Daru Noor or Sinopharm vaccine is randomly injected intramuscularly into the deltoid muscle of my arm.

I consider myself obligated and committed to comply with all of the following:

- In this study, I will receive a vaccine in the form of an intramuscular injection in the deltoid muscle of the arm, and after administering the vaccine, I will be monitored for 30 minutes to ensure my health fully.
- It is necessary to take blood samples for laboratory tests and perform periodic examinations during the study process. I agree to fully cooperate with the research team during this period.
- I will receive some forms regarding recording possible adverse events, which I will complete and submit on my next visit.
- I should not donate blood during the study. I should not participate in another clinical trial for the entire duration of this study. I will inform the research team if I have to use another medicine or vaccine or if hospitalized for any reason.

- All stages of the study, including the initial examination, taking samples for lab tests, receiving the study product, and subsequent tests and visits, will be performed at the study site.
- My cooperation with the study will be six months after the first injection.

6. The potential benefits of my participation in this study are as follows:
    - I will receive one injection of the covid-19 vaccine manufactured by Milad Daru Noor or Sinopharm vaccine in the intramuscular form.
    - I was instructed that if I were to be exposed to the virus in the future, I should not assume that because I had received the booster vaccine in this study, it would protect me 100% against the disease.
7. The possible harms of participating in this study are as follows:
    - Considering the approval of the safety of the vaccine produced by Milad Daro Noor in phases 1 and 2 and the approval of the Sinopharm vaccine by the World Health Organization and the Ministry of Health, the probability of harm and side effects following the vaccine is low. However, any drug or biological product may have side effects, including pain, redness, swelling, or bruising at the injection site, as well as mild symptoms such as fever, headache, fatigue, and body pain in the first 48 hours or most one week after the injection. These symptoms, if they occur, are expected to be mild, but there is also the possibility of moderate or severe symptoms.
8. If I don't want to participate in the study, I will not have any unpleasant consequences in terms of receiving health care services.
9. I know that all the information I provide to the research team is confidential. My name and identification details will not appear on any report. The study results will be published in aggregated form to protect the identity of the participants.
10. I know that the Research Ethics Committee can access my information to monitor the study's conduct and ensure that my rights have been observed.
11. I know that I will not bear any research project costs, including the cost of clinical examinations and laboratory tests. In the event of any harm done, all the expenses will be compensated by the study sponsor. The sponsor of this study is Milad Daroo Noor Company. I know that if, during and after the research, any physical and mental problems occur to me due to participating in this research, the treatment of complications and its costs and related compensation will be the sponsor's responsibility.
12. Phone numbers were given to me to answer, and I was told that whenever there is a problem or question related to participation in the mentioned research, to share with them and ask for guidance. In this regard, I agree that the phone calls made in connection with the questions and possible side effects of the vaccine with the doctors and clinical colleagues of the project will be recorded to record the research information accurately.
13. I know that if I have any problems or objections to those involved or the research process, I can contact the National Committee on Research Ethics and raise my concern orally or in writing at the following address:
    - National Ethics Committee at the Ministry of Health and Medical Education, floor 13, Block A, Simaye Iran Ave., between south Flamak and Zarafshan St., Shahrake-Gharb, Tehran, Iran. Tel: 0098 21 81455618.
14. This informed consent has two copies; one of the copies will be given to the participant.

I … hereby declare that I will observe the implementation of all the commitments made by the sponsor and oblige myself to do everything in my power to protect the safety and rights of the participants in this research.

Printed name of the chief investigator ________________________
Date & Signature of chief investigator __________________________

I … have read and understood this informed consent and am willing to participate in this research.
Printed name of the participant ________________________
Date & Signature of the participant __________________________
