# Supplementary material for: Comparison of the Safety and Immunogenicity of FAKHRAVAC and BBIBP-CorV Vaccines when Administrated as Booster Dose: A Parallel Two Arms, Randomized, Double Blind Clinical Trial
Source: Vaccines (Basel). 2022 Oct 26;10(11):1800. doi: 10.3390/vaccines10111800 (PMC9695457; doi:10.3390/vaccines10111800)
Supplement: Supplementary file 1 [file vaccines-10-01800-s001.zip › Supplementary File S4.pdf]

According to *Babaee et al.** study on adverse effects following COVID-19 vaccination in Iran, of 2653 participants who received two doses of COVID-19 vaccines, 55.56% reported adverse effects after the first dose and 35.7% after the second dose. Sputnik V caused the most adverse effects in 82.7% vaccine recipients, compared with 70.5% for AstraZeneca and 37.4% for the Sinopharm vaccine. Further, occurrence of adverse effects was significantly higher in participants with a history of COVID-19 infection who received the Sputnik V or AstraZeneca vaccine than those who either reported no history of infection or received Sinopharm vaccine (P = 0.001).

Comparison of Local adverse events in the current study (*Ahi et al*) with *Babaee et al* study.

	Sinopharm (Curent study) n=219	Fakhra (Curent study) n=216	Sinopharm (Babaee et al.)* n= 1564
Local adverse reactions	79 (36%)	103 (48%)	585 (37.4%)
Pain			
Grade 1	23 (11%)	27 (12.5 %)	
Grade 2	0 (0%)	1 (0.5 %)	
Tenderness			
Grade 1	40 (17%)	63 (29.5%)	
Redness			
Grade 1	3 (2%)	0 (0%)	
Swelling			
Grade 1	13 (6%)	12 (5.5%)	

*Babaee, E., Amirkafi, A., Tehrani-Banihashemi, A., SoleimanvandiAzar, N., Eshrati, B., Rampisheh, Z., Asadi-Aliabadi, M. and Nojomi, M., 2022. Adverse effects following COVID-19 vaccination in Iran. BMC Infectious Diseases, 22(1), 476.
